# Study on the cohesive shear characteristics and intrinsic modelling of the root–tailing soil interface of *Amorpha fruticosa*

**DOI:** 10.1038/s41598-022-15925-w

**Published:** 2022-07-12

**Authors:** Qing Chao Yang, Zhe Hao, Wenjing Cheng, Sheng You Lei, Ying Zhang, Da Teng, Qian Zhang, Xiao Ming Wang

**Affiliations:** 1 School of Highway, Chang’ an University, Xi’an, 710064 China; 2 School of Highway and Architecture, Shandong Transport Vocational College, Weifang, 261206 China; 3 College of Environmental Sciences, Liaoning University, Shenyang, 110036 China; 4 Nonferrous Geological Exploration and Research Institute Limited Liability Company, Shenyang, 110013 China

**Keywords:** Engineering, Civil engineering

## Abstract

To study the soil consolidation effect of shrub plant roots on tailings soil and to explore the frictional characteristics of plant roots on tailings soil, three experimental conditions of the root–soil interface were established by using a modified indoor direct shear instrument with binders such as liquid sodium silicate and cyanoacrylate to conduct direct shear frictional tests at the root–soil interface using the roots of the typical slope protection plant *Amorpha fruticosa*. The Gompertz improved curve model was established by using the relationship between shear stress and shear displacement and the trend of the root–soil interface parameter index. The results were compared between the improved Gompertz curve model and the Clough–Duncan hyperbolic model, and a two-factor coupled improved Gompertz interfacial intrinsic structure model with normal stress and cohesive strength factor was established. The results showed that the interface shear stress and shear displacement showed strain hardening characteristics at different normal pressures for cohesive strength ratios of 1.5 and 1.7 at the root–tailing soil interface. At a cohesive strength ratio of 1.6, strain-softening was observed from 100 to 300 kPa and strain hardening was observed at 400 kPa. The improved Gompertz curve model predicts the shear stress and shear displacement curves at the root–soil interface with different cohesive strengths more reasonably than the Clough–Duncan hyperbolic model, and the maximum accuracy can be improved by nearly 40%. The two-factor coupled improved Gompertz curve model can fit the shear stress versus shear displacement relationship at the *A. fruticosa* root–tailing soil interface.

## Introduction

Tailings ponds are the focus when studying the ecological impacts of mines^1,2^, and vegetation plays a vital role in controlling soil erosion and ensuring slope stability^3^. Plant roots penetrate into the soil and fully contact various media in the soil. There is a trend of mutual dislocation between roots and soil in the common process of deformation. This dislocation is resisted by the frictional resistance between roots and soil, which enhances the shear strength of the root–soil complex^4^ and improves the soil consolidation capacity. Therefore, the study of the frictional characteristics of root–soil contact surfaces is the key to the study of root–soil consolidation mechanisms^5^. In terms of root impact, Schwarz et al.^6^ proposed through field tests and indoor simulations that the friction between roots and soil is mainly the result of the joint action of adhesive friction, non-adhesive friction and shear friction. Ji et al.^7^ found that there was a positive power function correlation between the root diameter and the root–soil friction of *Pinus tabulaeformis*. Zhao et al.^8^ found that friction at the root–soil interface of Betula platyphylla roots in different altitudes and growth directions is also different, and the root diameter and altitude contribute more to the root–soil friction; however, they did not analyse the influence of the interface bonding effect. In terms of the constitutive model of the interface between geosynthetics and fillers, Anubhav et al.^9^ studied the shear stress displacement characteristics of a soil–geotextile interface and proposed a nonlinear Clough–Duncan hyperbolic constitutive model fitting strain softening. Esterhuizen et al.^10^ proposed using the Clough–Duncan hyperbolic model to fit the nonlinearity after the shear peak of the interface between clay and geotechnical materials and confirmed that the model fit the test data well. He et al.^11^ studied the shear mechanical properties of a loess–mortar interface and established the interface Clough–Duncan hyperbolic model before peak and considered the characteristics of strain softening. Gao et al.^12^ conducted a shear test of a loess–concrete interface and found that the modified Clough–Duncan hyperbolic model can better represent the contact between unsaturated loess and structures. However, little research has been done on using the Gompertz curve constitutive model^13^ for the root–soil interface.

In conclusion, although the constitutive model has been applied to the interface between geosynthetics and structures, research on the friction constitutive model of the plant root–soil interface is still relatively rare, especially involving the friction characteristics and their trend under the consideration of the bonding strength of the root–soil interface. In view of this, in this paper, the focus is on the ecological restoration area of the Waitoushan tailings dam in Liaoning Province. The dominant shrub *A. fruticosa* is taken as the test species, and the improved direct shear instrument is used to design three kinds of root–soil interface test conditions to carry out the direct shear friction test of the *A. fruticosa* root–tailing soil interface under different bonding strengths. The Gompertz improved curve model was established by using the relationship between shear stress and shear displacement and the trend of the root–soil interface parameter index. The Gompertz model is compared with the Clough–Duncan hyperbolic model. The improved Gompertz constitutive model of the two-factor coupling interface between normal stress and the bond strength factor is established. This is of great practical significance for the frictional testing of the root–soil interface and the study of constitutive models and provides theoretical support and practical application support for the ecological restoration and vegetation-based slope protection of tailings ponds.

## Materials and methods

### Material collection and preparation

**Figure 1:**
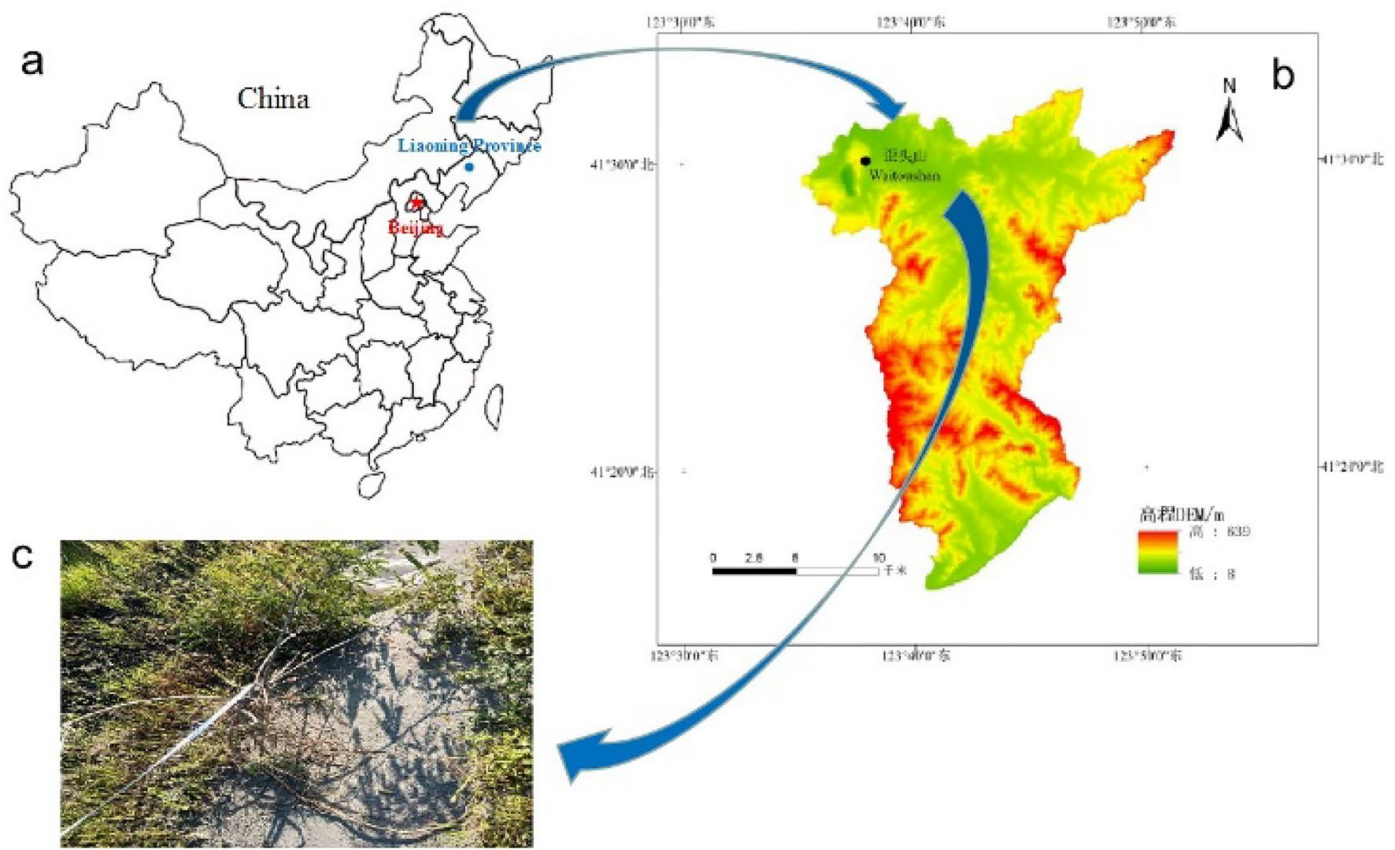
Site location and root system of tailing ponds in the study area. (**a**) Google Earth image snippets of the area (https://www.google.com/earth/) also show the location of the study area in Liaoning Province, China. (**b**) The topography map of Wai tou Mountain in Xi hu District, prepared by Qing chao Yang using ArcGIS ver. 10.8 (https://www.esri.com/), is based on the elevation data downloaded from the Geospatial Data Cloud (https://www.gscloud.cn/search). (**c**) The root images of *A. fruticosa* at sampling sites were recorded by camera.

**Figure 2:**
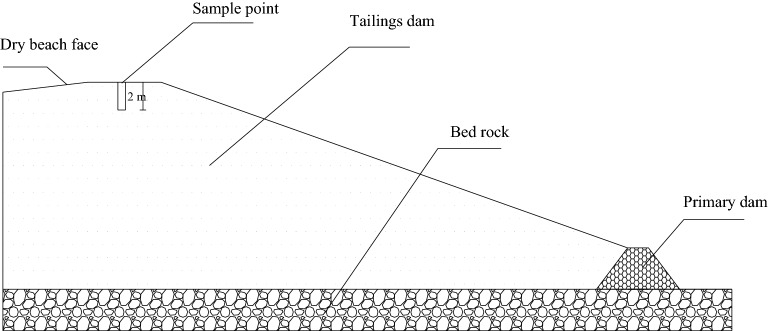
Schematic diagram of tailing sand sampling point.

**Figure 3:**
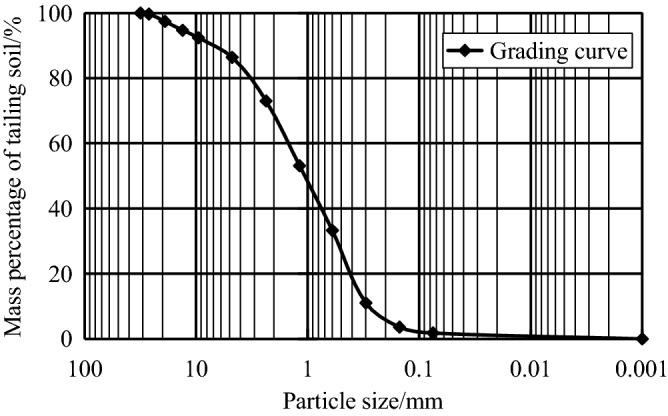
Accumulation curve of grain size gradation of tailings soil.

The sampling point of the test material is the valley-type tailings sand reservoir of the Waitoushan Iron Mine of the Benxi Iron and Steel Group. *A. fruticosa* is typical vegetation in the ecological restoration area on the outer slope of the tailings dam. After obtaining permission to use the research site from the Benxi Iron and Steel Group, the root system experiment was carried out with *A. fruticosa* as the representative plant. In August 2021, the dam slope platform of *A. fruticosa* planted for 4 years was selected as the experimental sampling area, and 30 healthy *A. fruticosa* plants were randomly selected on the platform. Referring to the methods of Sun^14^, Delory et al.^15^, Cornelissen et al.^16^ and Wang et al.^17^, the Liaoning Nonferrous Metals Survey and Research Institute, an authoritative CMA testing institution recognized by the national certification and accreditation supervision committee, was entrusted to test the plant height, crown width and ground diameter of *A. fruticosa*. According to CJ/T 24-2018, the industry standard for urban construction in the People's Republic of China, the ground diameter is the diameter of the main stem of the plant at 10 cm from the ground surface. The average plant height (122.6 ± 26.6 cm), crown width (125.5 ± 15.4 cm) and ground diameter (2.0 ± 0.5 mm) were used as the data of standard plants^18^. The complete excavation method^19^ was used for collection, which involves digging layer by layer and attempting to avoid mechanical damage to the root system. To ensure the collected specimens were representative, fresh roots with good growth conditions, undamaged epidermis and straight and uniform rootstocks were selected for the test, surface soil was removed from the roots with a brush. The plants were brought back to the laboratory in sealed bags, stored in a refrigerator at 4 °C^20^, and subjected to subsequent tests as soon as possible. Root systems with diameters ranging from 2 to 3 mm^18^ were used as test samples. The root systems of plants in the tailings ponds in the study area are shown in Fig. 1. Our study complies with the IUCN Policy Statement on Research Involving Species at Risk of Extinction and the Convention on the Trade in Endangered Species of Wild Fauna and Flora. The tailings soil sampled from the site is mostly tailings sand soil to prevent inconsistencies caused by debris such as dead leaves, fallen leaves, lumps and stones, and animal manure. This test tailings sand is sampled from the top of the tailings dam at a depth of 2 m. The test tailings sand sampling location is shown in Fig. 2 and was treated in accordance with the requirements of the GB/T 50123-2019 standard for geotechnical test methods. After drying for 8 h at 105 ± 5 °C, it was passed through a 2 mm aperture geotechnical sieve and sealed in a sealed bag for storage. Table 1 shows the basic physical parameters of tailings soil; Fig. 3 shows the cumulative curve of particle size grading of tailings sand.

**Table 1:** Basic physical parameters of tailings soil.

Natural water content	Natural density	Particle density	Natural porosity ratio	Plasticity index	Liquid index
w/%	ρ/g/cm^3^	G_s_	e_0_	W_P_	W_L_
9.6	1.94	2.71	0.69	14.3	0.15

### Test equipment and methods

#### Test equipment

**Figure 4:**
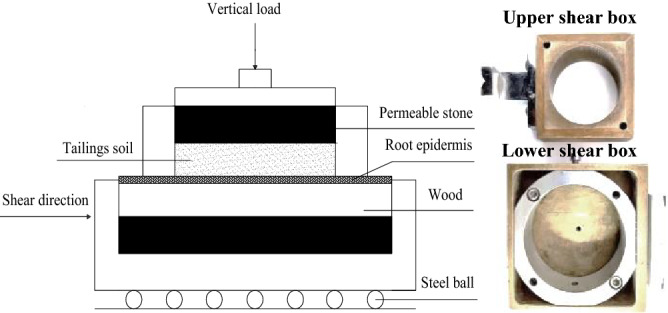
Schematic diagram of straight shear friction test.

To ensure the test conditions of adhesion strength at the root–soil interface, the lower shear box of EDJ-1 double-speed electric strain shear was improved to ensure the same contact area of the lower and upper shear boxes during the test, as shown in Fig. 4. The lower and upper shear box diameters are 80 mm and 61.8 mm, respectively, and the depth of the shear box is 20 mm. The upper shear box is loaded with permeable stone and tailings soil. The lower shear box is modified and loaded with a wood block and permeable stone. The wood block and the lower shear box are then combined tightly to ensure that the contact area between the tailings soil and the root surface on the wood block remains unchanged during the test.

#### Test method

The cohesive strength of the root–soil interface is derived from the shear strength exerted by the root–soil contact surface during shear and consists of two parts, interfacial cohesion and interfacial frictional resistance^21^. The interfacial cohesion is formed by chemicals with strong gelling effects (such as organic acids, mucilage, exoenzymes, etc.) secreted by the root surface during the growth of the root system, which forms interfacial chemical cohesion^21,22^ and interfacial soil cohesion^22^. Since there are limited studies of root–soil interfacial cohesive strength, previous experimental findings on root–soil interfacial cohesive strength and the commonly used binder materials are used (investigated by Xia et al.^21^, Guo et al.^23^, Zhang et al.^24^, Xia et al.^25^, Ge et al.^26^, Su et al.^27^, and Xing et al.^28^). The simulated results of liquid sodium silicate and cyanoacrylate root–soil interfacial cohesive strength were obtained by a direct shear friction experiment to determine the statistics of root–soil interfacial cohesive strength and can be used as the test interval (in the range of 1.1–28.3 kPa).

The interfacial adhesion was quantified based on the cohesion strength of 16.77 kPa at the interface between the *A. fruticosa* root and the tailing soil. A total of three different root–soil interfacial adhesion strength ratio gradients (1.5, 1.6 and 1.7) were determined. Three experimental conditions of the root–soil interface were determined to perform direct shear friction tests of the root–soil interface. A 1 cm thick permeable stone was placed in the lower box of the straight shear. A circular block of wood with root bark adhered to the permeable stone was placed on top of it. It was ensured that the root surface was flush with the surface of the lower shear box. The direction of the root axis was parallel to the shear direction, and the configured tailings specimen was placed on top of the block. Tailings soil has a natural moisture content of 9.6% (see Table 1); however, for the convenience of this test and in accordance with GB/T 50123-2019 “Standard for Geotechnical Test Methods”, a moisture content of 10% and density of 1.75 g/cm^3^ were used for tailings soil samples. Permeable stone was placed on the specimen, and the topmost end was placed on the pressure transfer plate. The test was conducted at 4 different vertical pressures of 100, 200, 300 and 400 kPa, with a shear displacement rate of 0.8 mm/min and a shear displacement of 6 mm. Four samples were tested in each group, and each group was repeated three times, with the tailings soil (CK) as the blank control. Details of the test sample information are shown in Table 2.

**Table 2:** Test sample information.

Test number	Type of test	Short form	Strength gradient
1	*A. fruticosa* root + tailings soil	RS	1.5
2	*A. fruticosa* root + liquid sodium silicate + tailings soil	NRS	1.6
3	*A. fruticosa* root + cyanoacrylate + tailings soil	GRS	1.7
4	Tailings soil	CK	–

### Voucher specimen information

Collection date: August 9, 2021. Collection number: 1-z. Collected by Xiaoming Wang, China. Collection site: Iron Mine Street, Crooked Head Mountain Town, Xihu District, Benxi City, Liaoning Province, China. Habitat: shrub.

### Identification information

Scientific name: Amorpha fruticosa, Identifier: Da Teng, Cross-reference identification method: Flora of China, Date of identification: December 20, 2021.

### Storage of information

The voucher specimens were stored in the botanical specimen room of the experimental centre of Liaoning Nonferrous Survey Research Institute, which is recognized by the National Accreditation and Supervision Commission as an authoritative CMA testing institution.

## Results

### Shear characteristics under different interfacial bonding strengths

#### The relationship between shear stress and shear displacement at the root–soil interface

**Figure 5:**
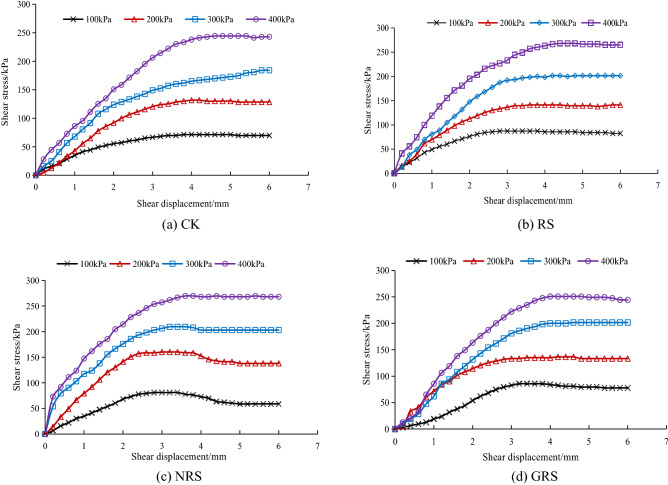
Relationship between shear stress and displacement of root–tailing soil and *A. fruticosa* under (**a**) CK, (**b**) RS, (**c**) NRS, and (**d**) GRS different interfacial bond strengths.

Figure 5 shows the relationship between the shear stress and shear displacement of the interface between *A. fruticosa* root and tailings soil under different interfacial bonding strengths.

As shown in Fig. 5, the peak shear stress increases when the normal pressure increases from 100 to 400 kPa, the CK peak shear stress increases from 71.42 to 244.40 kPa, and RS, NRS and GRS are larger than CK. Due to the increase in normal pressure in the shear process of the sample, the friction between soil particles in the shear zone increases^29^. The increase in the proportional gradient of CK, RS and GRS adhesive strength also increases the shear strength. The shear stress and shear displacement curves in Fig. 5a,b,d show strain hardening. The NRS interface exhibits a strain-softening type when the normal pressure increases from 100 to 300 kPa, as shown in Fig. 5c. This is because the bond strength of the root–soil interface is relatively strong and the soil structure is relatively intact. When the shear stress is less than the shear strength, the soil structure in the shear zone is subjected to a certain amount of shear^30^. When the shear stress exceeds the shear strength and begins to soften, the soil structure and the interfacial cementation state in the shear zone are destroyed, resulting in strain softening of the stress and displacement curves in the post peak strength phase. However, the friction between soil particles, which gradually increases with increasing normal pressure, leads to possible sliding of soil particles in the shear zone^31^ and a hardening phenomenon. Normal pressure and interfacial bonding strength have clear effects on the shape of the interfacial shear stress and shear displacement curve.

#### Shear strength index

**Figure 6:**
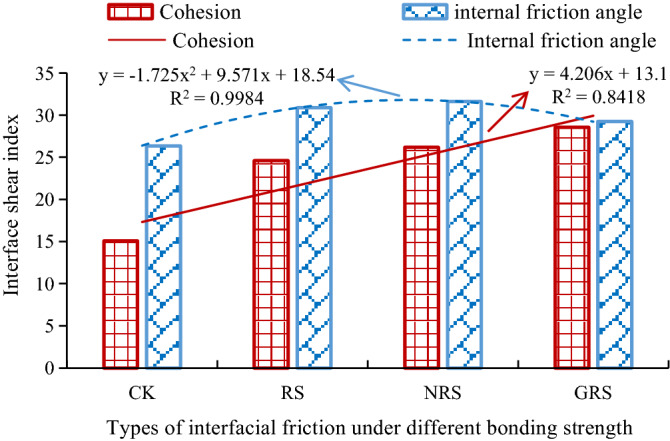
Relationship between shear index of the interface between *A. fruticosa* root and tailings soil.

Figure 6 shows the shear index relationship curve of the interface between *A. fruticosa* root and tailings soil. The cohesion of the interface increases with increasing bonding strength factor, and the internal friction angle increases first and then decreases, as shown in Fig. 6. At the same time, the shear strength indices of the interface are greater than those of the bare tailings sand interface. The minimum increase in RS cohesion was 63.2% higher than that in CK, and the maximum increase in GRS cohesion was 89.5% higher than that in CK.

#### Interface friction coefficient

**Figure 7:**
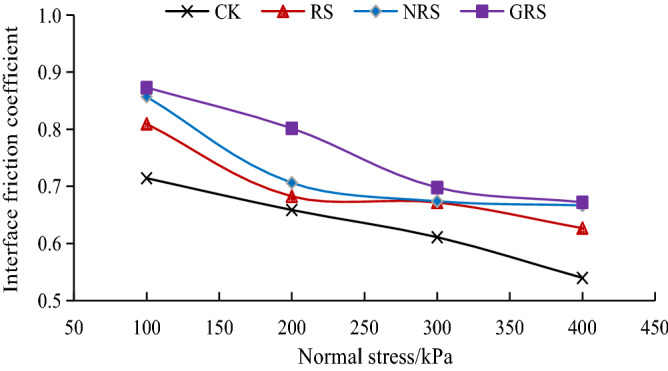
Variation curve of root–soil interface friction coefficient.

Figure 7 shows the variation curve of the friction coefficient at the interface of the *A. fruticosa* root and tailings soil. Under different conditions of interfacial bond strength, the interfacial friction coefficients of *A. fruticosa* root–tailed sandy soil were all greater than those of bare tailings soil, increasing from 3.62 to 24.54%. This shows that the root–soil interface bonding strength can effectively increase the friction coefficient between *A. fruticosa* root and tailings soil.

#### Equivalent shear stiffness of the interface

**Figure 8:**
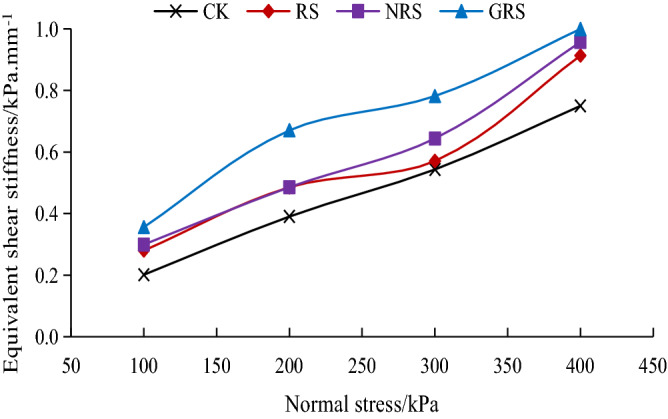
Variation curve of equivalent shear stiffness of root–soil interface.

Figure 8 shows the trend of the equivalent shear stiffness of the interface between *A. fruticosa* root and tailings soil. The equivalent shear stiffness of the *A. fruticosa* root–tailings soil interface is higher than that of the bare tailings soil interface. The equivalent shear stiffness of the interface showed different increases with increasing normal stress, from 5.11 to 77.09%. When the normal stress is 100 kPa, RS, NRS and GRS have the largest increase compared to other normal stresses (39.21%, 48.94% and 77.09%, respectively). At different positive stresses, GRS showed the largest increases of 77.09%, 71.58%, 43.90%, and 33.29%. Under different normal stresses, GRS increases the most, with increases of 77.09%, 71.58%, 43.90% and 33.29%. This shows that the root–soil interface bonding strength can effectively increase the equivalent shear stiffness of the *A. fruticosa* root–tailings soil interface.

## Interface constitutive model

### Improved Gompertz curve model

The expressions in the Gompertz prediction model mainly have the following forms^32–35^:

1 $$ Y = {\text{kce}}^{(b - cx)\exp ( - e(b - cx))} , $$

2 $$ Y = {\text{ke}}^{ - a\;\exp (b - ct)} , $$

3 $$ Y = {\text{ke}}^{ - a\;\exp ( - bt))} , $$

where $a,b,c,$ and $k$ are parameters;$t$ is the variation series; and $Y$ is the predicted value of the data.

The Gompertz curve model is a relatively complex nonlinear equation. The estimation of the nonlinear parameters $a,b,c,$ and $k$ is not only complex in calculation and not universal but also often causes large errors in the prediction results^36^, which affects the prediction accuracy of the model.

Therefore, an improved Gompertz curve model is proposed in this paper to fit the nonlinear relationship between shear stress and shear displacement at the *A. fruticosa* root–tailings soil interface.

4 $$ \tau = a\left( {e^{ - b\delta } - 1} \right), $$

where $\tau$ is the shear stress (kPa); $\delta$ is the shear displacement (mm);$a$ and ${\text{b}}$ are the fitting parameters for the straight shear friction test data.

To determine the shear stiffness of the shear stress versus displacement curve, the shear stiffness of the interface is obtained by deriving the shear displacement $\delta$ for Eq. (4).

5 $$ K_{st} = \frac{d\tau }{{d\delta }} = - abe^{b\delta } . $$

The initial shear strength is given by the following:

6 $$ K_{i} = \mathop {\lim }\limits_{\delta \to 0} \frac{d\tau }{{d\delta }} = - ab. $$

Peak shear stress of the model is given by:

7 $$ \tau_{ult} = \mathop {\lim }\limits_{\delta \to \infty } \tau = - a, $$

8 $$ {\text{R}}_{f} = \frac{{\tau_{p} }}{{\tau_{ult} }}. $$

The interface shear stiffness is obtained by substituting Eqs. (6) to (7) into Eq. (5).

9 $$ K_{st} = \frac{d\tau }{{d\delta }} = E_{i} \exp \left( { - \frac{{E_{i} Rf}}{{\tau_{p} }}\delta } \right). $$

From both Eqs. (7) and (8), the following can be obtained:

10 $$ K_{st} = K_{st} \left( {1 - \frac{{\tau R_{f} }}{{\tau_{p} }}} \right)^{2} , $$

11 $$ K_{i} = K\gamma_{W} \left( {\frac{{\sigma_{n} }}{{P_{a} }}} \right)^{n} , $$

where $K$ is the stiffness coefficient;$n$ is the stiffness index;$\gamma_{w}$ is the water weight (9.8 kN/m^3^); and $p_{a}$ is the standard atmospheric pressure (101.4 kPa).

Next, the logarithm of Eq. (11) is taken.

12 $$ \lg \left( {\frac{{K_{i} }}{{\gamma_{W} }}} \right) = \lg K + n\lg \left( {\frac{{\sigma_{n} }}{{{\text{P}}_{{\text{a}}} }}} \right). $$

Then, it can be seen that there is a linear relationship between ${{K_{i} } \mathord{\left/ {\vphantom {{K_{i} } {\gamma_{w} }}} \right. \kern-\nulldelimiterspace} {\gamma_{w} }}{ - }{{\sigma_{n} } \mathord{\left/ {\vphantom {{\sigma_{n} } {{\text{P}}_{{\text{a}}} }}} \right. \kern-\nulldelimiterspace} {{\text{P}}_{{\text{a}}} }}$ in the double logarithmic coordinate axis;$\lg K$ and $n$ are the intercept and slope of the corresponding lines.

The shear strength of the *A. fruticosa* root–tailings soil interface is proportional to the normal stress, and the interface shear strength conforms to the Mohr–Coulomb strength criterion^7,25^.

13 $$ \tau_{p} = c + \sigma_{n} \tan \varphi , $$

where $\tau_{p}$ is the interface shear strength (kPa);$\sigma_{n}$ is the normal stress (kPa);$c$ is the interface cohesion (kPa); and $\varphi$ is the interface internal friction angle (°).

Substituting Eq. (12) into Eq. (10), the interface shear stiffness can be derived.

14 $$ K_{st} = K\gamma_{W} \left( {\frac{{\sigma_{n} }}{{P_{a} }}} \right)^{n} \left( {1 - R_{f} \frac{\tau }{{\tau_{p} }}} \right). $$

### Model verification

**Figure 9:**
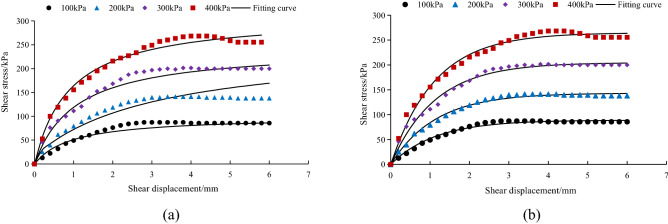
Fitting curve of RS interface model. (**a**) The Clough–Duncan curve model; (**b**) the improved Gompertz curve model.

**Figure 10:**
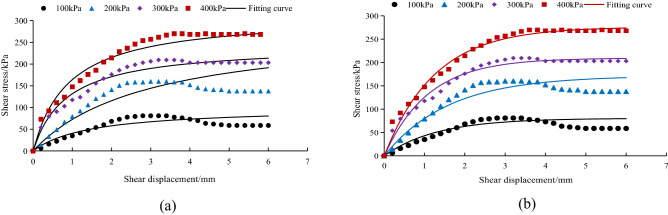
Fitting curve of NRS interface model. (**a**) The Clough–Duncan curve model; (**b**) the improved Gompertz curve model.

**Figure 11:**
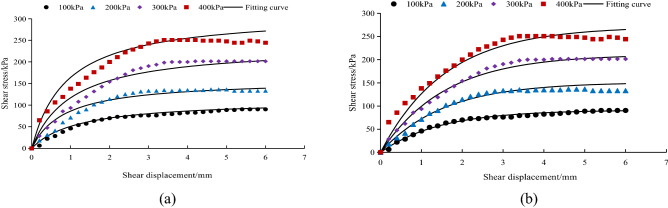
Fitting curve of GRS interface model. (**a**) The Clough–Duncan curve model; (**b**) the improved Gompertz curve model.

For the relationship between shear stress and shear displacement of the *A. fruticosa* root–tailings soil interface under different interface bonding strengths, the Clough–Duncan hyperbolic model^13,37^ and improved Gompertz curve model are used for fitting. The interface fitting parameters of the Clough–Duncan hyperbolic model and improved Gompertz curve model are listed in Table 3. The values of shear displacement and shear stress for the Clough–Duncan hyperbolic model and the modified Gompertz curve model at four normal pressures were calculated and plotted as curves for different interfacial bond strengths of RS, NRS and GRS using Table 3, as shown in Figs. 9, 10 and 11.

**Table 3:** Clough–Duncan model and improved Gompertz model fitting parameters.

Contact surface type	Model type	$\sigma_{n}$/kPa	$\tau_{ult}$/kPa	$\tau$/kPa	$R_{{\text{f}}}$ average	$K_{i}$/MPa	$K$	$n$
RS	Clough–Duncan	100	133.33	85.70	0.83	5.75	0.61	1.13
		200	151.52	136.48		13.16		
		300	237.98	201.55		22.40		
		400	268.82	250.74		26.04		
	Gompertz	100	89.11	85.70	0.96	78.69	7.91	0.80
		200	143.40	136.48		126.77		
		300	204.80	201.55		181.94		
		400	264.80	250.74		238.19		
NRS	Clough–Duncan	100	109.89	80.94	0.77	10.42	0.76	1.04
		200	169.49	160.29		15.94		
		300	311.72	209.48		24.39		
		400	383.73	269.79		29.25		
	Gompertz	100	80.45	80.94	0.98	61.76	6.25	0.96
		200	171.50	160.29		107.87		
		300	209.50	209.48		192.09		
		400	276.30	269.79		220.43		
GRS	Clough–Duncan	100	133.33	85.70	0.83	11.64	0.79	1.22
		200	151.52	136.48		21.01		
		300	237.98	201.55		28.57		
		400	268.82	250.74		34.55		
	Gompertz	100	89.78	85.70	0.96	62.99	6.39	0.82
		200	140.20	136.48		110.63		
		300	213.40	201.55		137.22		
		400	255.10	250.74		209.08

Under the interface of RS, NRS and GRS, the two models basically conform to the relationship between shear stress and shear displacement of the contact surface. The model curve increases with increasing shear displacement, but the degree of increase decreases continuously. After reaching the peak point it remains basically unchanged, and the trend is basically the same as that of the test point.

**Figure 12:**
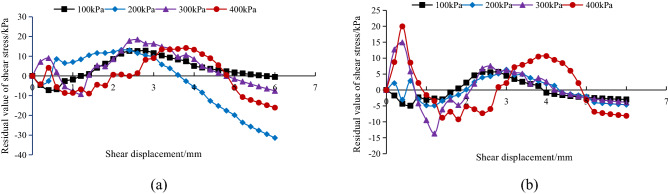
Residual shear stress of interface model under RS bonding strength. (**a**) The Clough–Duncan curve model; (**b**) the improved Gompertz curve model.

**Figure 13:**
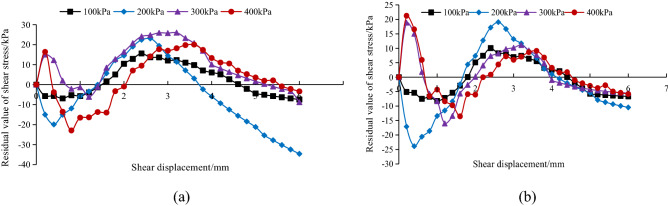
Residual shear stress of interface model under NRS bonding strength. (**a**) The Clough–Duncan curve model; (**b**) the improved Gompertz curve model.

**Figure 14:**
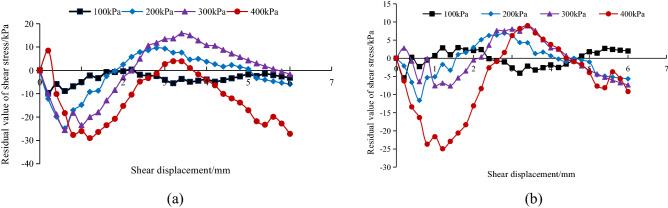
Residual shear stress of interface model under GRS bonding strength. (**a**) The Clough–Duncan curve model; (**b**) the improved Gompertz curve model.

To verify and compare the fitted relationship between the Clough-Duncan hyperbolic model and the modified Gompertz curve model, the difference between the experimental shear stress and the model shear stress value was used. The closer the difference is to zero, the better the model fit is. Figures 12, 13 and 14 show the relationship between the experimental shear stress minus the model shear stress value (i.e., the residual value of shear stress) and the shear displacement at the *A. fruticosa* root–tailings soil interface under different cohesive strength conditions.

Under the normal stress of the RS, NRS and GRS interfaces, the residual shear stress of the Clough–Duncan hyperbolic model is approximately 35 kPa, and the residual shear stress of the Gompertz curve model is approximately 25 kPa. The prediction is better using the Gompertz curve model, which shows a nearly 40% improvement in accuracy. When the RS interface normal stress is 400 kPa, the Clough–Duncan model provides better predictions than the Gompertz model, with shear stress residuals with a small difference of approximately 4 kPa; however, the other normal stresses are better predicted by the Gompertz model. The Clough–Duncan shear stress residual maximum is within approximately 35 kPa for the NRS interface normal stress of 200 kPa, while the Gompertz maximum is approximately 24 kPa, indicating that the Gompertz curve model provides better predictions. The Gompertz shear stress residual maximum value is approximately 25 kPa at the GRS interface normal stress of 200 kPa, while the Clough–Duncan maximum value is approximately 29 kPa, showing the superior predictions of the Gompertz curve model. In conclusion, the improved Gompertz model predicts the interface shear stress–shear displacement curves better than Clough–Duncan for the RS, NRS and GRS interfaces.

### Improved Gompertz model of the two-factor coupling root–soil interface

#### The improved Gompertz model of coupling normal stress and bond strength ratio

**Figure 15:**
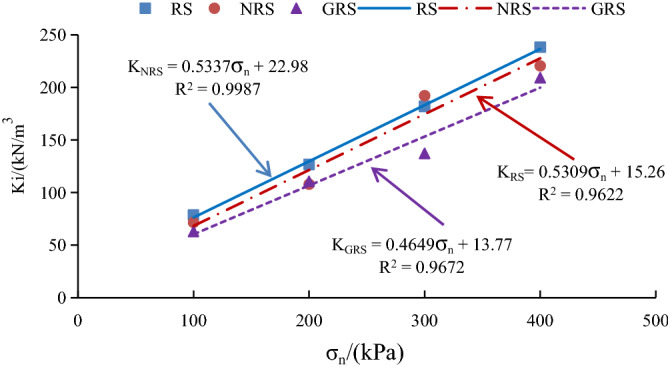
$K_{i} - \sigma_{n}$ linear fitting relationship.

**Figure 16:**
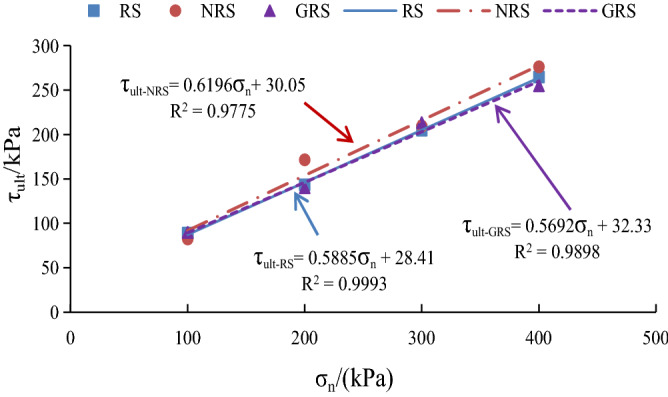
$\tau_{ult} - \sigma_{n}$ linear fitting relationship.

The initial shear stiffness $K_{i}$ and the correlation coefficient between ultimate shear stress $\tau_{ult}$ and normal stress $\sigma_{n}$ of *A. fruticosa* root–tailings soil under different interfacial bonding strengths obtained from the test reach accuracies of more than 0.9622 and 0.9775, respectively, showing a good linear fitting relationship. The fitting results are shown in Figs. 15 and 16.

Combining Figs. 15 and 16, the parameters q (22.98, 15.26, 13.77) and $n$(28.41, 30.05, 32.33) showed a linear pattern with the increase in the percentage of adhesive strength, and the parameters $p$(0.5337, 0.5309, 0.4649) and $m$(0.5885, 0.6196, 0.5692) showed a linear pattern with the percentage of adhesive strength. There was no obvious pattern in the variation with the ratio of adhesive strength. Therefore, the average values of parameters p and m were taken to obtain the relationship between $K_{i} ,\sigma_{n}$ and $a_{k}$ for the *A. fruticosa* root–tailings soil interface, as follows:

12 $$ K_{i} = 0.5\sigma_{n} - 46.1a_{k} + 91.1. $$

The relationship between $\tau_{ult} ,\sigma_{n}$ and $a_{k}$ is as follows:

13 $$ \tau_{ult} = 0.6\sigma_{n} + 19.6a_{k} - 1.1. $$

Substituting Eqs. (12) and (13) into Eq. (2), the expressions of the intrinsic model of the *A. fruticosa* root–tailings soil interface with coupled $\sigma_{n}$ and $a_{k}$ are collated.

14 $$ \tau = - (0.6\sigma_{n} + 19.6a_{k} - 1.1)\left( {e^{{ - \frac{{0.6\sigma_{n} + 19.6a_{k} - 1.1}}{{0.5\sigma_{n} - 46.1a_{k} + 91.1}}\delta }} - 1} \right). $$

#### Verification of the improved Gompertz model of the two-factor coupling root–soil interface

**Figure 17:**
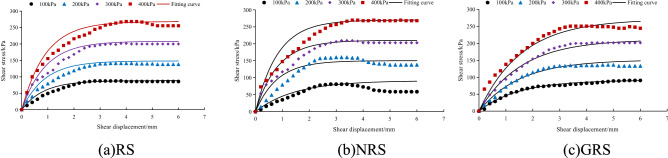
Fitted curves of experimental data and the improved Gompertz two-factor coupled model (**a**) RS, (**b**) NRS, and (**c**) GRS.

To verify the accuracy of the two-factor coupling constitutive model in fitting the relationship between shear stress and shear displacement at the interface between *A. fruticosa* root and tailings soil, the test data were fitted with the coupling model parameters and compared with the test data. Figure 17 shows the test data of interfacial shear stress displacement with different bonding strengths and the curve of the two-factor coupling improved Gompertz model.

Figure 17 shows that the improved Gompertz two-factor coupling model for the RS, NRS and GRS interfaces at different adhesive strengths agrees well with the experimental data, but there are still some deviations. This is because the shear stress versus shear displacement curves obtained by the direct shear test is not fully compatible with the modified Gompertz model. Meanwhile, according to the physical definition of the model parameters, it is known that the model parameters are related to the initial shear stiffness and shear strength. In the actual test, the errors caused by the test apparatus, the manual readings and other factors lead to a dispersion of the shear stress and displacement curves at the beginning of the stress phase and the final damage phase^38^, thus causing the difference between the initial shear stiffness and shear strength of the model curves and the test results. To accurately determine the reasonableness of the model, Table 4 shows the correlation coefficients (R^2^ values) between the model curves of shear stress and shear displacement at the interface between *A. fruticosa* root and tailings soil and the test results at different bonding strengths. Table 4 shows that the correlation coefficients of the fitting are above 0.87, indicating that the two-factor coupling constitutive model can, to a certain extent, predict the relationship between the shear stress and shear displacement of the interface between *A. fruticosa* root and tailings soil.

**Table 4:** Experimental data and model correlation coefficients.

$\sigma_{n}$/kPa	R^2^		
	RS	NRS	GRS
100	0.985	0.873	0.996
200	0.990	0.959	0.988
300	0.987	0.978	0.996
400	0.988	0.971	0.989

## Conclusion

In this paper, an improved indoor direct shear instrument is used to conduct direct shear friction tests on three root–soil bonding interfaces and analyse the bonding shear characteristics and constitutive model of the *A. fruticosa* root–tailings soil interface.

1. Using the improved direct shear apparatus, the direct shear friction test of the *A. fruticosa* root–tailings soil interface under different bonding strengths is carried out. The normal pressure and interfacial bonding strength have clear effects on the shape of the interfacial shear stress and shear displacement curve. When the interfacial bonding strength ratio is 1.5 and 1.7, the shear stress and shear displacement curves of the *A. fruticosa* root–tailings soil interface under different normal pressures show strain hardening. When the interfacial bonding strength ratio is 1.6, it shows a strain softening type when the normal pressure is from 100 to 300 kPa and a strain hardening type when the normal pressure is 400 kPa.
2. The cohesion of the *A. fruticosa* root–tailings soil interface increases with increasing bonding strength factor ratio, and the internal friction angle first increases and then decreases. The shear strength indices of the interface between *A. fruticosa* roots and tailings soil are higher than those of bare tailings soil. The root–soil interface adhesion can effectively improve the parameters such as interface cohesion, interface friction coefficient and equivalent shear stiffness of *A. fruticosa*.
3. The improved Gompertz curve model is established. For the interface between *A. fruticosa* root and tailings soil under different bonding strengths, the prediction of the improved Gompertz curve model of the interface shear stress and shear displacement curve is more reasonable than that of the Clough–Duncan hyperbolic model; the maximum accuracy can be improved by nearly 40%.
4. The fitting under different interfacial bonding strengths shows that the $K_{i} - \sigma_{n}$ and $\tau_{ult} - \sigma_{n}$ of the *A. fruticosa* root–tailings soil interface are linear. Accordingly, the interface bonding strength ratio $a_{k}$ is introduced, the $\sigma_{n} - a_{k}$ two-factor coupling interface improved Gompertz constitutive model is established, and the coupling model parameters are proposed. The correlation coefficients between the calculated results and the experimental data are more than 0.87, indicating that the two-factor coupling constitutive model can effectively predict the relationship between shear stress and shear displacement at the interface of *A. fruticosa* root and tailings soil.

## Data Availability

The datasets generated during and/or analysed during the current study are available from the corresponding author on reasonable request.
